# Income differences in partial life expectancy between ages 35 and 64 from 1988 to 2017: the contribution of living arrangements

**DOI:** 10.1093/eurpub/ckac159

**Published:** 2022-11-15

**Authors:** Jade Knop, Pekka Martikainen, Hanna Remes, Lasse Tarkiainen

**Affiliations:** Population Research Unit, Faculty of Social Sciences, University of Helsinki, Helsinki, Finland; Population Research Unit, Faculty of Social Sciences, University of Helsinki, Helsinki, Finland; Population Research Unit, Faculty of Social Sciences, University of Helsinki, Helsinki, Finland; Population Research Unit, Faculty of Social Sciences, University of Helsinki, Helsinki, Finland

## Abstract

**Background:**

Socioeconomic differences in mortality among the working-age population have increased in several high-income countries. The aim of this study was to assess whether changes in the living arrangement composition of income groups have contributed to changing income differences in life expectancy during the past 30 years.

**Methods:**

We used Finnish register data covering the total population to calculate partial life expectancies between ages 35 and 64 by income quartile in 1988–2017. The contribution of living arrangements to these differences was assessed by direct standardization. Decomposition methods were used to determine the extent of life expectancy differences due to external (accidental, violent and alcohol-related) causes of death.

**Results:**

The life expectancy gap between the highest and lowest income quartile increased until 2003–07, but decreased thereafter. The contribution of living arrangements to these differences remained mostly stable: 36–39% among men and 15–23% among women. Those living without children consistently showed the greatest life expectancy differences by income. External causes of death significantly contributed to income differences in life expectancy.

**Conclusions:**

The living arrangement composition of income groups explained part of the differences in life expectancy, but not their changes. Our results on the contribution of external causes of death imply that both the persistent income gradient in mortality as well as the mortality disparities by living arrangements are at least partially related to similar selection or causal mechanisms.

## Introduction

Increasing socioeconomic differences in mortality among the working-age population have been documented in several high-income countries in recent decades.^1–4^ Inequalities may be particularly stark between income groups,^5^ since the indicator allows for the identification of the most economically disadvantaged. Besides economic disadvantage, health-related selection, health behaviours and sociodemographic factors have been suggested to explain income differences in mortality. Previous research suggests that part, but not all, of the income–mortality association may be explained by pre-existing health status.^6–8^ Behavioural arguments are supported by findings indicating a significant contribution of alcohol- and smoking-related mortality to socioeconomic life expectancy differences, as well as changes therein.^9–11^ The contribution of sociodemographic factors, such as living arrangements and family characteristics, has been less studied, although these factors seem to account for some of the observed socioeconomic differences in mortality.^3^^,^^12^^,^^13^

Simultaneously, a related but largely separate line of research has documented increasing mortality differences by marital status, mainly stemming from a sharp and unparalleled decline in mortality among the married in the late 20th century.^14^^,^^15^ These disparities have been attributed to selection into and out of marriage based on health status^16–19^ or other, e.g. socioeconomic, characteristics^18^ as well as health-promoting material^19–22^ and psychosocial resources shared between spouses, such as social support and control of health behaviours.^19^^,^^22–25^ Increases in accidental, violent and alcohol-related causes of death among the non-married suggest that changes in health-related behaviours may have played a role in growing marital status differences in mortality in 1976–2000.^14^ Mortality differences by marital status have also been found to attenuate after controlling for socioeconomic status,^14^^,^^26^^,^^27^ but their increase over time does not seem to be explained by changes in socioeconomic factors, such as education or occupation.^14^^,^^28^ Since marital status alone does not capture the relationships and resources within families and households, a more comprehensive classification of living arrangements has been proposed to examine differences in health and mortality.^26^^,^^27^ The relevance of household composition is further demonstrated by findings suggesting that living alone and the presence of a partner may in fact be stronger independent predictors of mortality differences than marital status *per se*,^29^ and that childlessness and non-residential parenthood seem to be related to excess mortality among the working-age population.^27^^,^^30^^,^^31^

Research on the joint contribution of socioeconomic status and living arrangements to mortality differences has been scarce. As controlling for household characteristics has been found to attenuate mortality differences by socioeconomic status,^3^^,^^12^^,^^13^ and *vice versa*,^14^^,^^26^^,^^27^ there is reason to believe that similar mechanisms, such as health-related selection and health behaviours, may induce inequalities along both axes. Persons with poor health are more likely to become unemployed,^32^^,^^33^ leading to lower income, and less likely to marry,^16^^,^^17^ which may be reflected in mortality differences across income and marital status groups. Also, mortality trends and differences by income, marital status and living arrangements tend to be especially pronounced for behaviour-related i.e. accidental, violent and alcohol- and smoking-related causes of death.^9^^,^^11^^,^^14^^,^^27^ Furthermore, a causal link between socioeconomic and family characteristics cannot be excluded: a lack of socioeconomic resources may reduce the chances of union formation,^34^ while certain family transitions, such as divorce, may entail a loss of income.^35^

In recent decades, unmarried cohabitation, single parenting and living alone have become increasingly common, while the proportion of married couples and families with children has declined in many countries.^36–38^ It is noteworthy that increases have taken place in living arrangements associated with poorer health and higher mortality, and in some cases, lower socioeconomic status.^27^^,^^39^ Whether recent changes in living arrangements have implications for socioeconomic mortality differences depends on whether selection into different living arrangements or the strength of the association between household characteristics and mortality have changed. If, e.g. living alone is increasingly concentrated among those with low income, this ‘double burden’ may increase mortality differences by income. Given the interrelations between living arrangements, socioeconomic status and health, it is important to consider how changes in living arrangements may be reflected in trends in socioeconomic mortality differences.

To our knowledge, there are no previous studies examining the contribution of living arrangements to income differences in life expectancy in the working-age population. This study had four specific aims: (i) to quantify changes in income differences in partial life expectancy between ages 35 and 64 during the past 30 years, (ii) to assess how the contribution of living arrangements to the differences between the highest and lowest income group has changed, (iii) to examine how life expectancy differences by income differ across living arrangement groups and (iv) to determine to what extent these differences were attributable to accidental, violent and alcohol-related causes of death.

## Methods

The data used in this study cover all persons aged 35–64 years residing in Finland between 1988 and 2017. Statistics Finland linked data from various registers to death records using personal identification codes. Register linkage was authorized by data protection authorities in Statistics Finland (permission TK-53-1490-18). The study population was followed for mortality in 1988–2017, stratifying person-years and deaths by sex, 5-year age group, income quartile and living arrangements measured at the end of the year prior to follow-up. Person-years and deaths were aggregated for six 5-year periods (1988–92 through 2013–17). Those reaching the age of 65 years during follow-up were censored at the end of the year, and those turning 35 years were included in the data from the next year onwards. Individuals who emigrated or were institutionalized were censored at the end of the year. Altogether, the dataset included 63.1 million person-years and 288 185 deaths.

Information on income was derived from the registers of the Finnish Tax Administration and the Social Insurance Institution. We used the annual sum of taxable income of all household members, consisting of wages, capital income and taxable income transfers. Household composition was taken into account by dividing the total household income by the square root of the number of household members.^40^ Cut-off points for income quartiles were calculated within ages 35–64 separately for each year and sex.

Individuals’ living arrangements were determined according to information on their marital and cohabitation status, family composition and household size. Seven groups were formed: married with/without children, cohabiting with/without children, single parent, living alone and other. Statistics Finland defined cohabiters as persons who were living in the same dwelling, unmarried, at least 18 years of age, of different sex, not siblings and had an age difference of no more than 15 years. The group of others covered adults living with their parents or someone else than their partner or children, and those whose living arrangements were unknown.

Directly age-standardized mortality rates were calculated using the total male and female population aged 35–64 years in 1988–92 and 2013–17 as the standard population. Age-specific mortality rates by 5-year age group, sex, income quartile and living arrangements were used to obtain abridged period life tables with partial life expectancies between ages 35 and 64, and their confidence intervals, for each income quartile, 5-year period and living arrangement group. Life expectancy differences between the highest and lowest income quartile were decomposed by cause of death in each living arrangement group using Arriaga’s method.^41^^,^^42^ Causes of death were classified as either internal or external using the harmonized cause-of-death classification of Statistics Finland, based on the International Classification of Diseases (ICD). The external category encompassed accidents, violence and suicides, and alcohol-attributable diseases and poisoning (see table 2 for specific ICD-10 codes). All other causes of death, as well as unknown causes (0.33%), were included in the internal category. To assess the contribution of living arrangements to differences in partial life expectancy between the highest and lowest income quartile, we calculated adjusted mortality rates for the lowest income quartile. This was done by weighting the age-specific mortality rates of the lowest income quartile so that the living arrangement distribution of each 5-year age group corresponded to that of the highest quartile.

**Table 2:** Partial life expectancy between ages 35 and 64 by living arrangements among men and women in the lowest and highest income quartiles in 1988–92 and 2013–17

	1988–92								2013–17								
							Difference highest–lowest								Difference highest–lowest		Change in difference highest–lowest (years)
Living arrangements	Highest quartile	(95% CI)	Lowest quartile	(95% CI)	All	(95% CI)	Years	**External causes of death (%)** ^a^	Highest quartile	(95% CI)	Lowest quartile	(95% CI)	All	(95% CI)	Years	**External causes of death (%)** ^a^	
Men																	
Married with children	28.9	(28.8–28.9)	28.1	(28.0–28.2)	28.5	(28.5–28.6)	0.8	34	29.6	(29.6–29.7)	29.2	(29.1–29.2)	29.5	(29.5–29.5)	0.5	33	−0.3
Married without children	28.7	(28.6–28.8)	26.4	(26.0–26.8)	27.9	(27.8–28.0)	2.3	50	29.5	(29.4–29.6)	28.0	(27.7–28.3)	29.1	(29.1–29.2)	1.5	61	−0.8
Cohabiting with children	28.5	(28.2–28.8)	27.1	(26.7–27.5)	27.7	(27.5–27.9)	1.4	37	29.7	(29.6–29.8)	29.0	(28.9–29.2)	29.4	(29.4–29.5)	0.6	39	−0.7
Cohabiting without children	28.2	(28.0–28.4)	24.2	(23.8–24.7)	26.9	(26.7–27.0)	4.0	56	29.6	(29.5–29.6)	27.5	(27.2–27.8)	29.0	(28.9–29.1)	2.1	51	−1.9
Single parent	28.2	(27.6–28.5)	26.6	(26.2–27.0)	27.3	(27.1–27.5)	1.6	59	29.6	*(29.0–29.9)*	28.4	(28.2–28.6)	29.0	(28.9–29.1)	1.2	41	−0.5
Alone	28.0	(27.8–28.1)	23.2	(23.0–23.4)	25.4	(25.3–25.5)	4.7	53	29.3	(29.2–29.3)	26.1	(26.0–26.2)	27.6	(27.5–27.6)	3.2	52	−1.6
Other	27.0	(26.8–27.3)	23.6	(23.4–23.7)	25.0	(24.9–25.1)	3.5	48	29.0	(28.9–29.2)	26.2	(26.1–26.4)	27.4	(27.3–27.4)	2.8	39	−0.7
All	28.6	(28.6–28.7)	25.7	(25.6–25.8)	27.6	(27.6–27.6)	3.0	47	29.5	(29.5–29.6)	27.3	(27.2–27.3)	28.8	(28.8–28.8)	2.3	48	−0.7
Women																	
Married with children	29.4	(29.4–29.4)	29.2	(29.1–29.3)	29.3	(29.3–29.3)	0.2	9	29.8	(29.7–29.8)	29.4	(29.4–29.5)	29.7	(29.6–29.7)	0.3	21	0.1
Married without children	29.3	(29.2–29.4)	28.1	(27.8–28.4)	28.9	(28.9–29.0)	1.2	42	29.6	(29.6–29.7)	28.6	(28.4–28.8)	29.4	(29.4–29.5)	1.0	63	−0.2
Cohabiting with children	29.4	(29.1–29.6)	28.7	(28.3–29.0)	29.0	(28.9–29.1)	0.7	49	29.8	*(29.7–29.8)*	29.4	(29.2–29.5)	29.6	(29.6–29.7)	0.4	32	−0.3
Cohabiting without children	29.2	(29.0–29.3)	25.8	(25.3–26.3)	28.2	(28.1–28.4)	3.4	60	29.7	(29.6–29.7)	28.0	(27.7–28.3)	29.3	(29.3–29.4)	1.7	42	−1.7
Single parent	29.3	(29.0–29.4)	28.9	(28.8–29.0)	29.0	(29.0–29.1)	0.4	26	29.8	*(29.6–29.9)*	29.4	(29.3–29.4)	29.5	(29.5–29.5)	0.4	38	0.0
Alone	29.4	(29.3–29.5)	27.2	(27.0–27.4)	28.6	(28.5–28.6)	2.2	44	29.7	(29.6–29.8)	28.1	(27.9–28.2)	28.9	(28.9–29.0)	1.6	43	−0.6
Other	28.8	(28.5–29.1)	26.1	(25.9–26.4)	27.4	(27.2–27.5)	2.6	25	29.4	*(29.2–29.6)*	26.6	(26.4–26.9)	28.0	(27.9–28.1)	2.8	19	0.1
All	29.4	(29.3–29.4)	28.4	(28.3–28.4)	29.0	(29.0–29.0)	1.0	34	29.7	(29.7–29.7)	28.7	(28.7–28.8)	29.4	(29.4–29.4)	1.0	34	0.0

The 95% confidence intervals were calculated using Chiang’s I method. For some of the highest quartile groups with no deaths within 5-year age group, the variance of the conditional probability of death was calculated assuming one death in the age group. These 95% CIs are in italics.

a ICD-10 codes F10, G312, G4051, G621, G721, I426, K292, K70, K860, K8600, O354, P043, X45, V01–X44, X46–X59, X85–Y89, X60–X84 and Y870.

## Results

A clear income gradient in mortality was present for men and women in both the first and last period of follow-up, with particularly high death rates in the lowest income quartile (table 1). High mortality was also observed among those living alone and in ‘other’ living arrangements. There were notable changes in living arrangement distributions between periods, with the share of married persons living with children decreasing and the proportion of cohabiters and those living alone increasing.

**Table 1:** Age-adjusted mortality rates and distribution of person-years by income and living arrangements among men and women aged 35–64 in 1988–92 and 2013–17

	Men				Women			
	1988–92		2013–17		1988–92		2013–17	
Income group	**Mortality rate** ^a^	Person-years (%)	**Mortality rate** ^a^	Person-years (%)	**Mortality rate** ^a^	Person-years (%)	**Mortality rate** ^a^	Person-years (%)
First quartile (highest)	4.9	25	1.7	25	2.2	25	1.0	25
Second quartile	6.5	25	2.6	25	2.6	25	1.3	25
Third quartile	8.5	25	4.0	25	3.3	25	1.9	25
Fourth quartile (lowest)	14.9	25	10.1	25	5.2	25	4.6	25
Living arrangements								
Married with children	5.9	49	2.1	32	2.6	45	1.2	30
Married without children	7.2	20	3.0	21	3.2	22	1.8	24
Cohabiting with children	8.9	3	2.2	7	3.4	3	1.7	7
Cohabiting without children	11.3	4	3.8	8	5.3	3	2.3	7
Single parent	10.4	2	3.6	2	3.4	9	2.0	10
Alone	16.4	12	8.7	22	4.5	14	3.4	19
Other	18.2	10	10.5	8	8.4	4	7.4	3
All	8.8	100	4.6	100	3.4	100	2.2	100
*n* (1000s)^b^	38	4856	26	5296	16	4888	13	5271

a Age-adjusted mortality rates per 1000 person-years. Standard population: combined population of men and women in 1988–92 and 2013–17.

b Number of deaths/person-years in thousands.

**Figure 1:**
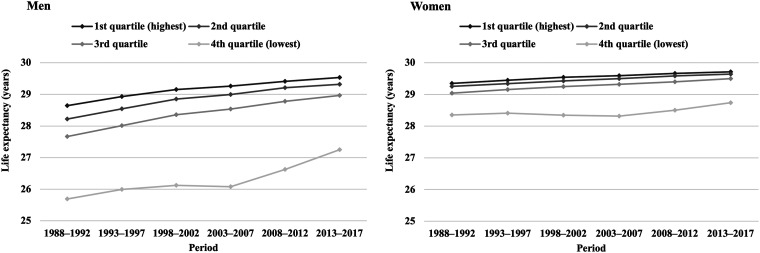
Partial life expectancy between ages 35 and 64 by income group among men and women in 1988–2017

**Figure 2:**
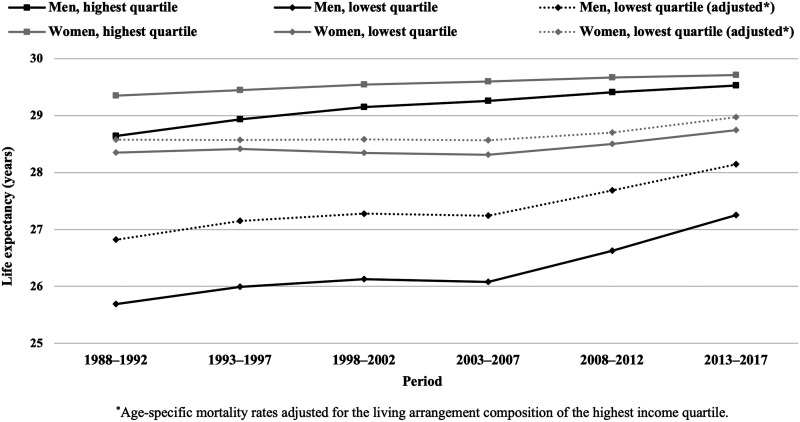
Adjusted* and unadjusted partial life expectancy between ages 35 and 64 among men and women in the highest and lowest income quartiles in 1988–2017

Partial life expectancy between ages 35 and 64 increased in all income quartiles during the study period (figure 1). For men, the increase was largest in the lowest income quartile (1.6 years, vs. 0.9 years in the highest quartile), whereas for women, gains in life expectancy were similar in all income quartiles (0.4–0.5 years). Among both sexes, the gap between the highest and lowest income quartile widened until 2003–07, and narrowed thereafter. As a result, the 1-year gap among women remained, while the difference decreased from 3.0 to 2.3 years among men during the 30-year follow-up. The contribution of living arrangements to the life expectancy gap was mostly stable throughout the study period. Adjusting for living arrangements explained 36–39% and 15–23% of the difference between the top and bottom quartiles among men and women, respectively (figure 2).

Partial life expectancy increased in all living arrangement groups in both the highest and lowest income quartile during the study period (table 2). Income differences in partial life expectancy were smaller among women than men in each living arrangement group. Differences between the highest and lowest quartile were greatest among those living alone and cohabiting without children. The gap in life expectancy narrowed the most in these living arrangement groups due to substantial life expectancy increases in the lowest income quartile. By 2013–17, life expectancy differences among men living alone and cohabiting without children had decreased to 3.2 and 2.1 years, respectively. The corresponding differences among women were 1.6 and 1.7 years. There were only minor changes among women in other living arrangement groups. Among men, increases in life expectancy were greater in the lowest income quartile in all living arrangement groups. Those married with children showed the smallest income differences in life expectancy among men in both periods, matched by cohabiting men with children in 2013–17, with respective differences of 0.5 and 0.6 years. Among women, the gap in life expectancy was smallest among those living with children, whether married, cohabiting or single parents, at 0.3, 0.4 and 0.4 years, respectively, in 2013–17. Among both sexes, life expectancy differences between living arrangement groups were much smaller within the highest income group, further converging during the study period.

The contribution of accidental, violent and alcohol-related causes of death to income differences in life expectancy remained stable between the first and last period at 47–48% among men and 34% among women (table 2). However, the contribution of external causes varied between 21% and 63% across living arrangement groups, so that these causes tended to contribute more among groups with larger life expectancy differences by income. Living with children was associated with smaller contributions of external causes of death.

## Discussion

### Principal findings

Income differences in partial life expectancy between ages 35 and 64 increased from 1988–92 to 2003–07, followed by a decrease from 2003–07 to 2013–17. For men, these developments resulted in a net decrease in life expectancy differences, while differences remained largely unchanged for women. Despite changes in the distribution of living arrangements, their contribution to life expectancy differences by income remained stable throughout the study period. Living arrangements explained 36–39% and 15–23% of the life expectancy gap between the highest and lowest income quartile among men and women, respectively.

The life expectancy gap between the highest and lowest income quartile was largest among cohabiters without children and those living alone, but also narrowed the most in these groups. The gap was smallest among those living with children, whether married, cohabiting or single parents, especially among women. By 2013–17, life expectancy differences between living arrangement groups had almost disappeared in the highest income quartile. The contribution of external causes of death to income differences in life expectancy remained stable during the study period at 47–48% among men and 34% among women. This contribution was greater among those living without children.

### Interpretation of the results

Increasing income differences in life expectancy have been documented in several high-income countries in recent decades. Substantial increases have been reported in the USA,^1^^,^^43^ while more moderate ones have been observed in Canada,^44^ Denmark^43^ and Norway.^2^ These results mostly apply to differences in life expectancy at all adult ages, although increasing inequalities have been observed especially among the working-age population.^9^ In contrast to these prior findings, we observed a decrease in life expectancy differences by income from 2003–07 to 2013–17. Existing evidence suggests that this decline originates from a reduction in alcohol-related mortality in the lowest income group, particularly among men.^45^

Previous studies on socioeconomic mortality differences have rarely considered the contribution of living arrangements, although family and household characteristics are known to be socially patterned and to predict mortality outcomes especially in the working-age population.^14^^,^^46^^,^^47^ Our analyses revealed that life expectancy differences by income tended to be greatest in living arrangement groups associated with lower life expectancy, such as living alone or cohabiting without children. The magnitude of these differences implied a significant life expectancy disadvantage, a ‘double burden’, for persons living alone or cohabiting without children in the lowest income quartile. Despite the largest relative disadvantage, life expectancy differences by income were reduced the most in these living arrangement groups during the study period. These changes may be associated with relative improvements in the status of these groups. Living alone and cohabiting without children increased notably during the study period and, with the exception of women living alone, these groups also experienced the greatest absolute gains in life expectancy.

We observed substantial attenuations in life expectancy differences by income after adjusting for living arrangements. These attenuations reflect the fact that persons with lower income tend to live in less favourable living arrangements more often, e.g. are more likely to be unmarried and live alone (Supplementary table S1). Our results suggest that living with children may have mortality-protective effects among the working-age population, which is in line with previous findings.^27^^,^^29^^,^^30^ Furthermore, this advantage seems to extend to single parents, especially single mothers, despite previous studies indicating poorer health among them.^40^^,^^48^^,^^49^ Life expectancy differences by income were smaller among those living with children, as was the contribution of accidental, violent and alcohol-related causes of death to these disparities. These findings suggest that parenthood may be particularly protective among those with low income. Life expectancy differences by income, as well as the contribution of living arrangements to these differences, were greater among men than women. Men in the lowest income quartile are more likely than their higher-income or female counterparts to live in unfavourable arrangements with respect to mortality, as living without a partner is more common among those with lower income, and child custody mainly remains a female responsibility. In 2013–17, 42% of men and 33% of women in the lowest income quartile were living alone, whereas 24% of women and only 4% of men in the lowest income quartile were living in single parent households (Supplementary table S1).

The notion that similar causal or selection mechanisms may underlie mortality differences by income and living arrangements is supported by our results on the contribution of accidental, violent and alcohol-related causes of death to income differences in life expectancy. The excess mortality of the lowest income group relative to the highest one was explained by external causes of death to a greater extent in living arrangements associated with a lower life expectancy and greater life expectancy differences by income. However, these results do not allow us to disentangle causal and selection effects to determine to which extent mortality differences are due to the influence of socioeconomic and household characteristics on health behaviours, or behaviour-related selection into income and living arrangement groups. In addition, more upstream social determinants of mortality, such as education and occupational class, as well as relationship trajectories preceding current living arrangements are likely underlying factors behind our findings. We encourage future studies to investigate the contribution of these determinants to income and living arrangement differences in mortality across the life course.

### Methodological considerations

The register data used in the study provide reliable measurement free of self-report bias, covering the entire Finnish population over three decades with virtually no loss to follow-up and avoiding problems related to self-reported income. Individuals with zero income comprised only 0.3% of the study population in the first and last 5-year period, as we measured income at the household level, including taxable social security benefits. As an indicator of socioeconomic status, income quantiles allow for keeping the proportions of groups constant over time and are therefore well suited for the study of temporal changes in mortality. To test the sensitivity of our results to the categorization of income, alternative analyses were performed using income quintiles (Supplementary table S2). The results were highly consistent with those obtained with income quartiles, with life expectancy differences between extreme groups increasing 0.0–0.4 years across living arrangement categories.

Statistics Finland’s classification of cohabitation used in the study inevitably results in the misclassification of some individuals, since the definition does not cover couples with a large age difference or of the same sex, but might classify persons not living in a relationship, such as flatmates, as cohabiting partners. However, this is unlikely to considerably affect the results, as the share of persons classified as cohabiters resembles that obtained from nationally representative survey data in 2017, although survey estimates were somewhat higher among women aged 30–39 and men aged 40–49.^50^

## Conclusions

Income differences in partial life expectancy in working age (35–64 years) increased until 2003–07, but decreased towards 2013–17. The contribution of living arrangements to differences between the top and bottom income quartiles was substantial and remained stable throughout the study period. Differences by income were greater among those living in households without children, particularly men living alone, and were largely related to accidental, violent and alcohol-related causes of death. Our results on the importance of these causes of death imply that both the persistent income gradient in mortality as well as the mortality disparities by living arrangements are at least partially related to similar selection or causal mechanisms.

## Supplementary data

Supplementary data are available at *EURPUB* online.

## Funding

This work was supported by grants 308247 and 345219 from the Academy of Finland and grant 101019329 from the European Research Council.

*Conflicts of interest*: None declared.

## Supplementary Material

ckac159_Supplementary_Data

## Data Availability

Due to data protection regulations of the national register holders providing the data, they are not allowed to make the data available to third parties. Interested researchers have the possibility to obtain data access by contacting Statistics Finland (https://www.stat.fi/tup/mikroaineistot/index_en.html). Little is known about whether family and household characteristics are associated with increasing socioeconomic mortality differences in the working-age population. We used Finnish register data covering all persons aged 35–64 between 1988 and 2017. The contribution of living arrangements to income differences in life expectancy was substantial and mostly stable over the study period. Accidental, violent and alcohol-related causes of death contributed more to income differences in life expectancy in living arrangement groups where these differences were more pronounced, such as persons living without children. Our results suggest that similar causal or selection mechanisms, related to e.g. health behaviours, may underlie mortality inequalities across both income and living arrangement groups.
