# Supplementary material for: Income differences in partial life expectancy between ages 35 and 64 from 1988 to 2017: the contribution of living arrangements
Source: Eur J Public Health. 2022 Nov 15;33(1):13–9. doi: 10.1093/eurpub/ckac159 (PMC10263265; doi:10.1093/eurpub/ckac159)
Supplement: ckac159_Supplementary_Data [file ckac159_Supplementary_Data.docx]

| **Supplementary Table S1.** Distribution (%) of person-years by living arrangements among men and women aged 35–64 in the highest and lowest income quartiles in 1988-1992 and 2013-2017. | | | | | | | | | | | |
| --- | --- | --- | --- | --- | --- | --- | --- | --- | --- | --- | --- |
|  |  |  |  |  |  |  |  |  |  |  |  |
|  | **Men** | | | | |  | **Women** | | | | |
|  | **1988–1992** | |  | **2013–2017** | |  | **1988–1992** | |  | **2013–2017** | |
| **Living arrangements** | **Highest quartile** | **Lowest quartile** |  | **Highest quartile** | **Lowest quartile** |  | **Highest quartile** | **Lowest quartile** |  | **Highest quartile** | **Lowest quartile** |
| Married with children | 53 | 35 |  | 34 | 20 |  | 54 | 27 |  | 37 | 17 |
| Married without children | 26 | 16 |  | 33 | 10 |  | 27 | 20 |  | 37 | 12 |
| Cohabiting with children | 3 | 3 |  | 5 | 6 |  | 3 | 2 |  | 6 | 4 |
| Cohabiting without children | 5 | 3 |  | 10 | 5 |  | 5 | 3 |  | 10 | 4 |
| Single parent | 1 | 2 |  | 1 | 4 |  | 3 | 18 |  | 2 | 24 |
| Alone | 7 | 22 |  | 12 | 42 |  | 6 | 24 |  | 7 | 33 |
| Other | 5 | 18 |  | 4 | 14 |  | 2 | 7 |  | 2 | 6 |
| All | 100 | 100 |  | 100 | 100 |  | 100 | 100 |  | 100 | 100 |

| **Supplementary Table S2.** Partial life expectancy between ages 35–64 by living arrangements among men and women in the lowest and highest income quintiles in 1988–1992 and 2013–2017. | | | | | | | | | | | | | | |
| --- | --- | --- | --- | --- | --- | --- | --- | --- | --- | --- | --- | --- | --- | --- |
|  |  |  |  |  |  |  |  |  |  |  |  |  |  |  |
|  |  |  |  |  |  |  |  |  |  |  |  |  | |  |
|  | **1988–1992** | | | |  | **2013–2017** | | | |  |  |  | |  |
| **Living arrangements** | **Highest quintile** | **Lowest quintile** | **All** | **Difference highest-lowest (years)** |  | **Highest quintile** | **Lowest quintile** | **All** | **Difference highest-lowest (years)** |  | **Change in difference highest-lowest (years)** | |  |  |
| **Men** |  |  |  |  |  |  |  |  |  |  |  | |  |  |
| Married with children | 28,9 | 28,1 | 28.5 | 0,9 |  | 29,6 | 29,2 | 29.5 | 0,5 |  | -0,4 | |  |  |
| Married without children | 28,7 | 26,2 | 27.9 | 2,5 |  | 29,5 | 27,9 | 29.1 | 1,6 |  | -0,9 | |  |  |
| Cohabiting with children | 28,5 | 27,2 | 27.7 | 1,3 |  | 29,7 | 29,0 | 29.4 | 0,7 |  | -0,6 | |  |  |
| Cohabiting without children | 28,2 | 24,0 | 26.9 | 4,2 |  | 29,6 | 27,1 | 29.0 | 2,4 |  | -1,8 | |  |  |
| Single parent | 28,0 | 26,5 | 27.3 | 1,5 |  | 29,4 | 28,3 | 29.0 | 1,1 |  | -0,4 | |  |  |
| Alone | 28,0 | 23,0 | 25.4 | 5,0 |  | 29,3 | 25,9 | 27.6 | 3,4 |  | -1,6 | |  |  |
| Other | 27,1 | 23,4 | 25.0 | 3,7 |  | 29,0 | 26,0 | 27.4 | 3,0 |  | -0,7 | |  |  |
| All | 28,7 | 25,4 | 27.6 | 3,3 |  | 29,5 | 26,9 | 28.8 | 2,6 |  | -0,7 | |  |  |
| **Women** |  |  |  |  |  |  |  |  |  |  |  | |  |  |
| Married with children | 29,4 | 29,2 | 29.3 | 0,2 |  | 29,8 | 29,4 | 29.7 | 0,3 |  | 0,1 | |  |  |
| Married without children | 29,3 | 27,9 | 28.9 | 1,4 |  | 29,7 | 28,4 | 29.4 | 1,2 |  | -0,2 | |  |  |
| Cohabiting with children | 29,3 | 28,7 | 29.0 | 0,6 |  | 29,8 | 29,3 | 29.6 | 0,5 |  | -0,1 | |  |  |
| Cohabiting without children | 29,2 | 25,6 | 28.2 | 3,7 |  | 29,7 | 27,7 | 29.3 | 2,0 |  | -1,7 | |  |  |
| Single parent | 29,2 | 28,9 | 29.0 | 0,3 |  | 29,7 | 29,3 | 29.5 | 0,4 |  | 0,1 | |  |  |
| Alone | 29,5 | 26,9 | 28.6 | 2,6 |  | 29,8 | 27,9 | 28.9 | 1,9 |  | -0,7 | |  |  |
| Other | 28,7 | 26,0 | 27.4 | 2,7 |  | 29,3 | 26,4 | 28.0 | 2,9 |  | 0,2 | |  |  |
| All | 29,4 | 28,2 | 29.0 | 1,2 |  | 29,7 | 28,6 | 29.4 | 1,1 |  | 0,0 | |  |  |

## Additional references

41. OECD. Framework for integrated analysis. In: OECD Framework for Statistics on the Distribution of Household Income, Consumption and Wealth. Paris: OECD Publishing, 2013; p. 171–92. http://dx.doi.org/10.1787/9789264194830-en (June 2013, date last accessed).

42. Arriaga EE. Measuring and explaining the change in life expectancies. *Demography* 1984;21:83–96.

43. Auger N, Feuillet P, Martel S, Lo E, Barry AD, Harper S. Mortality inequality in populations with equal life expectancy: Arriaga’s decomposition method in SAS, Stata, and Excel. *Ann Epidemiol* 2014;24:575–80.

44. Dahl G, Kreiner CT, Nielsen TH, Serena BL. Linking Changes in Inequality in Life Expectancy and Mortality: Evidence from Denmark and the United States. Cambridge, MA: National Bureau of Economic Research, 2020.

45. Milligan K, Schirle T. The Evolution of Longevity: Evidence from Canada. Cambridge, MA: National Bureau of Economic Research, 2018.

46. Tarkiainen L, Martikainen P, Peltonen R, Remes H. The gap in life expectancy between socioeconomic groups has not widened during the 2010s. Suom Lääkärilehti. 2017;72(9):588–693.

47. Jalovaara M, Fasang AE. Are there gender differences in family trajectories by education in Finland? *Demogr Res* 2015;33:1241–56.

48. Davis MA, Neuhaus JM, Moritz DJ, Segal MR. Living Arrangements and Survival Among Middle-Aged and Older Adults in the NHANES I Epidemiologic Follow-Up Study. *Am J Public Health Wash* 1992;82:401–6.

49. Benzeval M. The self-reported health status of lone parents. *Soc Sci Med* 1998;46:1337–53.

50. Whitehead M, Burström B, Diderichsen F. Social policies and the pathways to inequalities in health: a comparative analysis of lone mothers in Britain and Sweden. *Soc Sci Med* 2000;50:255–70.

51. Koponen P, Borodulin K, Lundqvist A, Sääksjärvi K, Koskinen S. Health, functional capacity and welfare in Finland – FinHealth 2017 study. Helsinki: Finnish Institute for Health and Welfare, 2018.
